# Incidence of diabetes retinopathy and determinants of time to diabetes retinopathy among diabetes patients at Tikur Anbessa Hospital, Ethiopia: a retrospective follow up study

**DOI:** 10.1186/s13104-018-3660-7

**Published:** 2018-08-02

**Authors:** Tabot Keskis Azeze, Malede Mequanent Sisay, Ejigu Gebeye Zeleke

**Affiliations:** 1 Ethiopian Postal Service Enterprise, Addis Ababa, Ethiopia; 2 Department of Epidemiology and Biostatistics, Institute of Public Health, College of Medicine and Health Sciences, University of Gondar, Gondar, Ethiopia

**Keywords:** Complication of diabetes mellitus, Diabetes retinopathy, Ethiopia, Incidence, Determinants

## Abstract

**Objective:**

Data regarding diabetes retinopathy and associated factors are currently lacking in Ethiopia. The study aims to determine the incidence and determinants of time to diabetes retinopathy among diabetes mellitus patients at Tikur Anbessa Specialized Hospital, Addis Ababa, Ethiopia.

**Results:**

The incidence of diabetes retinopathy is a rapidly growing burden of disease in Ethiopia. The incidence rate of diabetes retinopathy was 2.65 (95% CI 2. 54, 4.05) per 1000 person-years observation. Moreover, 70 (18.57%, 95% CI 14.63, 22.5) DM patients developed diabetes retinopathy. The median time was 74.07 months (with IQR 53.60, 89.88). Male sex (AHR = 1.94, 95% CI = 1.10, 3.39), type 2 DM (AHR = 4.01, 95% CI = 1.34, 12.00), creatinine (AHR = 2.59, 95% CI = 1.91, 3.52), borderline triglyceride (AHR = 2.87, 95% CI 1.33, 6.21) and high triglyceride levels (AHR = 2.59, 95% CI = 1.31, 4.97) were positively correlated factors to diabetes retinopathy occurrence. Multisectoral, population-based approaches are needed to reduce type 2 DM complications.

## Introduction

Diabetes mellitus (DM) is an important public health problem throughout the world and its prevalence has been steadily increasing over the past few decades [1]. Diabetes retinopathy (DR) is one of the life-threatening complications and is the most common cause of acquired blindness in adults [1–3].

Studies done in different countries showed that the incidence of DR was geographically heterogeneous, including in low and middle-income countries [4–15]. According to a systematic review, DR affects 30.2–31.6% of diabetes patients in Africa [16]. Specifically, the prevalence of DR is growing most rapidly in Sub-Saharan Africa countries imposing a large economic burden on these countries [17–24].

The most common risk factors related to DR are socio-demographic characteristics such as age, sex, body mass index (BMI), hypertension, poor glycemic control, type 2 diabetes mellitus (T2DM), blood pressure (BP), cholesterol level and time since diabetes diagnosis [22, 25–31]. However, the importance of the above factors varies between studies.

Several studies in Ethiopia have shown that the presence and severity of complications related to DR are steadily increasing cause of premature death and disability [19, 22, 32]. The incidence of DR has been well documented and risk factors are known in developed countries. However, studies regarding the incidence of related to diabetes retinopathy are scarce in Ethiopia and among the greater Africa continent in general. Therefore, the aim of this study was to identify the risk factors leading to DR among patients with diabetes at Tikur Anbessa specialized Hospital (TAH), Addis Ababa, Ethiopia.

## Main text

### Methods

An institution-based retrospective follow up study was conducted at Tikur Anbessa Hospital, the largest referral public and teaching hospital in the country, located in the capital city of Ethiopia, Addis Ababa. The hospital receives approximately 370,000–400,000 patients referred from across Ethiopia yearly [32]. The hospital has had an established diabetes clinic since 1994. Nearly 10,000 patients have enrolled the clinic due to diabetes related retinopathy disease and over 869 DM patients visited the hospital in 2017.

Patients newly diagnosed with type 1 or type 2 DM from January 2009 to May 2017 were considered for this study. Those who had at least one follow up visit and adults (aged ≥ 15 years) were included. The required sample size (373) was determined using a single population formula $n\, = \,\frac{{Z_{\alpha /2}^{2} \,*\,p(1 - p)}}{{d^{2} }}$, where n is the sample size; z is the value of standard normal distribution corresponding to a significant level of α of 5%, which is 1.96; d is the margin of error which was taken as 5% and p is the estimated proportion of DR among diabetes patients 41.4% [33]. Data were collected by standard data extraction checklist, entered using Epi Info 7 and exported to STATA 14 software for analysis. The outcome variable was time to DR due to DM after dilated eye examination using tonometry and ophthalmologic instruments. Diabetic retinopathy diagnosis includes a medical history, an ophthalmic examination and screening with retinal photographs, and regular follow up. In short, diabetes retinopathy was defined by both direct and indirect ophthalmoscopy assessments done by retinal specialists confirmed by fundus photography. Diabetes retinopathy occurs when blood vessels in the retina and optic nerve are damaged using dilated eye exam (drops are placed in the eyes to widen or dilate the pupils) using a special magnifying lens. Diabetes retinopathy apparat if there is leaking blood in retinal vessels, Pale, fatty deposits on the retina indicating leaking blood vessels. The incidence of DR was determined from the start of diagnosis date of DM until the last follow-up visit or known date of DR occurrence.

We used Cox regression survival model, since time to DR is non-negative values often exhibiting highly skewed distributions and censors [34]. Variables in the bi-variable proportional hazard model with a p-value below 2% were subsequently included in the multivariable analysis. The parsimonious model was selected using the log likelihood test and Akaike’s information criterion (AIC). Schoenfeld residual and Cox-Snell residual plot were used to check the model’s assumptions. The adjusted hazard ratio (AHR) with their respective 95% confidence intervals (CI) were reported to show the strength of the associations.

### Results

A total of 377 adult diabetes patients were included in the final analysis. Of these, 194 (51.5%) were females and 315 (83.6%) resided in Addis Ababa. The average age at initiation was 45 years (SD ± 15.76). Approximately, 174 (47.7%) had normal body mass index.

About 266 (70.6%) of the study participants presented with type 2 DM and 263 (69.8%) had no history of hypertension. More than half, 206 (54.6%) were taking oral drug regimens for diabetes treatment. Furthermore, 151 (40%) and 242 (64.2%) had normal systolic and diastolic BPs, respectively. Nearly two-thirds, 252 (66.8%) had very high fasting blood sugar and only 148 (45.5%) had desirable triglyceride levels. The majority of the DM patients, 267 (82.2%) had acceptable low-density lipoprotein levels. Whereas the median creatinine was 0.96 mg/dl (IQR 0.8–1.2) (Table 1).

**Table 1:** Demographic, clinical and physiological characteristics of DM patients at Tikur Anbessa Hospital, Addis Ababa, Ethiopia, 2009–2017

Variables	Category	DR	Censored	Frequency	Percent
Type of DM	Type 1	13	98	111	29.4
	Type 2	57	209	266	70.6
Sex	Male	26	157	183	48.5
	Female	44	144	194	51.5
Residence	Addis Ababa	59	256	315	83.6
	Out of Addis Ababa	11	51	62	16.4
BMI	Underweight	5	35	40	11
	Normal	35	139	174	47.7
	Overweight	16	90	106	29
	Obese	10	35	45	12.3
Regimen of DM	Insulin	29	142	171	45.4
	Oral	41	165	206	54.6
Hypertension	Yes	25	89	114	30.2
	No	45	218	263	69.8
Systolic BP (mmHg)	≤ 110	19	105	124	32.9
	110–130	30	121	151	40
	≥ 130	21	81	102	27.1
Diastolic BP (mmHg)	≤ 60	12	51	63	16.7
	60–80	48	194	242	64.2
	≥ 80	10	62	72	19.1
Fasting blood sugar (mg/dl)	< 70	5	10	15	4
	70–100	9	30	39	10.3
	100–125	13	57	70	18.8
	≥ 126	46	206	252	66.8
Total cholesterol (mg/dl)	< 200	37	163	200	61.5
	200–239	10	36	69	21.2
	> 240	20	36	56	17.3
Triglyceride (mg/dl)	< 150	23	125	148	45.5
	150–199	12	38	50	16.5
	≥ 200	32	95	127	39.1
Low density lipoprotein (mg/dl)	< 150	52	215	267	82.2
	150–190	2	13	15	4.6
	≥ 190	13	30	43	13.2
High density lipoprotein (mg/dl)	≤ 40	28	98	126	38.8
	41–59	26	100	126	38.8
	≥ 60	13	60	73	22.4

From the total, 70 (18.57%, 95% CI 14.63, 22.50) developed diabetes retinopathy. The incidence rate was 2.65 (95% CI 2.54, 4.05) per 1000 adult-months observation with a total follow-up time of 26,384.9 adult-months observation. Moreover, the incidence rate was 21.4 and 11.7% among Type 2 and Type 1 DM, respectively.

The study participants were followed for a minimum of one and a maximum of 108 months after their initial DM diagnosis with 74 (IQR 53.60, 89.88) months median follow-up time.

From multivariable Weibull cox regression analysis, the expected hazard ratio of DR was 1.94 times higher in male DM patients than in female ones while keeping other covariates constant (AHR = 1.94; 95% CI 1.10, 3.39). Furthermore, when the baseline creatinine level of DM patients increased by a single mg/dl, the hazard ratio of developing DR increased by 1.78 (AHR = 1.78:95% CI; 1.58, 2.01). High triglyceride levels among patients with DM increases their likelihood developing DR by 2.59 than those who have desirable triglyceride levels (AHR = 2.59, 95% CI 1.33, 6.21). Similarly, the expected hazard ratio of DR was 2.87 times higher among DM patients who had borderline high triglycerides compared to DM patients who had desirable triglyceride levels (AHR = 2.87; 95% CI 1.31, 4.97). In regards to complication status, patients with type 2 DM are 4 times more likely to develop DR compared to type 1 DM patients (AHR = 4.01; 95% CI 1.34, 12.0) (Table 2).

**Table 2:** Multiple Weibull regression model analysis of time to diabetes retinopathy occurrence among diabetes mellitus patients in 2009–2017 at Tikur Anbessa Hospital, Addis Ababa, Ethiopia

Variables	Category	CHR [95% CI]	AHR [95% CI]
Age		1.02 (1.012, 1.03)	0.99 (0.97, 1.02)
Creatinine		*1.78 (1.58, 2.01)*	*2.59 (1.91, 3.52)*
Sex	Female	*1*	*1*
	Male	*1.79 (1.35, 2.38)*	*1.94 (1.10, 3.39)*
Type of DM	Type 1	*1*	*1*
	Type 2	*2.71 (1.9, 3.86)*	*4.01 (1.34, 12.00)*
Systolic BP	Normal	1	1
	Low	0.67 (0.47, 0.93)	0.98 (0.52, 1.94)
	High	1.06 (0.78, 1.47)	0.66 (0.35, 1.23)
Fasting blood sugar	Normal	1	1
	Hypo	0.27 (0.08, 0.91)	0.15 (0.10, 0.51)
	High	0.99 (0.6, 1.63)	0.59 (0.24, 1.48)
	Very high	0.91 (0.59, 1.37)	0.62 (0.29, 1.30)
Cholesterol status	Desirable	1	1
	High	0.83 (0.55, 1.25)	0.93 (0.44, 1.97)
	Very high	2.39 (1.74, 3.29)	1.55 (0.76, 3.21)
Triglycerides	Desirable	*1*	*1*
	Borderline	*2.25 (1.49, 3.39)*	*2.87 (1.33, 6.21)*
	High	*2.28 (2.04, 3.86)*	*2.59 (1.31, 4.97)*
Low-density lipoprotein	Desirable	1	1
	Borderline high	1.5 (1.07, 2.19)	1.78 (0.83, 3.82)
	High	0.73 (0.32, 1.66)	0.64 (0.14, 2.93)
DM regimen	Insulin	1	1
	Oral	1.55 (1.18, 2.06)	0.71 (0.39, 1.29)
Hypertension	No	1	1
	Yes	1.43 (0.88, 2.34)	1.51 (0.48, 4.74)

### Discussion

This study attempted to determine the incidence of diabetes retinopathy and the risk factors among DM patients at Tikur Anbessa specialized hospital, Addis Ababa, Ethiopia. The proportion of DR was 18.57% with 2.65 per 1000 adult-months observation. The study showed a higher incidence of DR than studies done in Bangladesh and China [13, 29]. This inconsistency might be due to the difference in health care systems and the quality of care given to diabetes patients. However, the incidence rate in this study was lower than that of a study done at Arbaminch general hospital, Ethiopia [35]. This difference might also be due to the data collection period included in the study. Our study included 9 years of follow up data, whereas the Arba Minch study used 3 years follow up data. Moreover, has been a general health care improvement in Ethiopia recently [36].

The study identified that among DM patient’s males had a shorter time than females to develop diabetes retinopathy. This result is in line with that of a study conducted in China [29, 37]. This might be due to the fact that neuroretinal function is more abnormal in adult males than in adult females [38]. However, a study conducted in Ethiopia [39] showed that males had a lower risk of developing microvascular complications of DM than females.

This study showed that the hazard of diabetes retinopathy was increased with increasing creatinine levels. This result is similar to that of a study done in New Zealand [40, 41]. One reason for this may be that increasing creatinine values may decrease retinal fiber layer thickness [42].

The risk of developing DR among Type 2 DM patients was higher than among Type 1 DM patients in this study. The result is similar to the study conducted at Ayder referral hospital in Ethiopia and noted that type 2 DM patients had a shorter time to developing microvascular DM complications than Type 1 DM patients [39]. This may be due to the older age of most of the Type 2 patients and their inability to support themselves to receive appropriate eye screening services [43]. Furthermore, since the disease process of Type 2 DM is more gradual than Type 1 DM, these patients may seek health services later in the disease course. In contrast, a study conducted in Spain found that type 1 diabetes patients had a higher risk to develop DR than type 2 [44]. The most likely explanation for this difference may relate to the study design, shorter follow-up period or smaller sample size as well as early screening.

The result in this study indicated that DM patients who had borderline high (150–199 mg/dl) and high (200–499 mg/dl) triglyceride (TG) levels had a lower likelihood of DR than DM patients who had desirable (< 150 mg/dl) TG level. This finding is consistent with the study findings in France [45] and Birmingham [46]. This is because raised TG levels increase blood viscosity, which in turn leads to the formation of hard exudates. In addition, TG incorporates into the cell membrane, altering its fluidity and permeability which ultimately may results in hemorrhages [47].

### Conclusion

The incidence rate of diabetes retinopathy remains a high public health concern as the median time to developing diabetes retinopathy was relatively short. Health care workers should bear in mind that in addition to using drugs, consistent and proper triglyceride and creatinine level monitoring, as well as early screening and treatment of diabetes complications would be an essential part of DM care and delaying the onset of DR.

## Limitations

One of the major limitations of the current study is the absence of institutional and behavioral factors, which may underestimate the effects and individual variations in the development of diabetes retinopathy. Secondly, Even though the study was conducted in the largest national referral center in which many adults come from around the country for DM follow up, the findings could not be representative of the adult diabetes population in the country due to possible selection bias.

Because of the retrospective nature of the study, some important complications of diabetes that had a significant association with DR, like chronic kidney disease were missed since we used chart review to obtain the data.

## Abbreviations

AIC: Akaike’s information criterion
BMI: body mass index
Cox-PH: Cox-proportional hazard
DBP: diastolic blood pressure
DR: diabetes retinopathy
DM: diabetes mellitus
DME: diabetes mellitus edema
HbA1c: glycated hemoglobina1c
HDL: high-density lipid profile
HR: hazard ratios
LDL: low-density lipid profile
NPDR: non-proliferative diabetes retinopathy
PDR: proliferative diabetes retinopathy
SBP: systolic blood pressure
TAH: Tikur Anbessa Hospital
TC: total cholesterol
TG: triglyceride
IDF: International Diabetes Federation
VEGF: vascular endothelial growth factor
VTDR: vision-threatening diabetes retinopathy
WESDR: Wisconsin Epidemiological Study of Diabetes Retinopathy
WHO: World Health Organization
